# Supplementary material for: Pigment epithelium-derived factor promotes peritoneal dissemination of ovarian cancer through induction of immunosuppressive macrophages
Source: Commun Biol. 2022 Sep 2;5:904. doi: 10.1038/s42003-022-03837-4 (PMC9440245; doi:10.1038/s42003-022-03837-4)

Supplementary Information

Supplementary Figure 1.

Supplementary Figure 2.

Supplementary Figure 3.

Supplementary Figure 4.

Supplementary Figure 5.

Supplementary Figure 6.

Supplementary Table 1.

Supplementary Table 2.

Supplementary Table 3.

Gating information

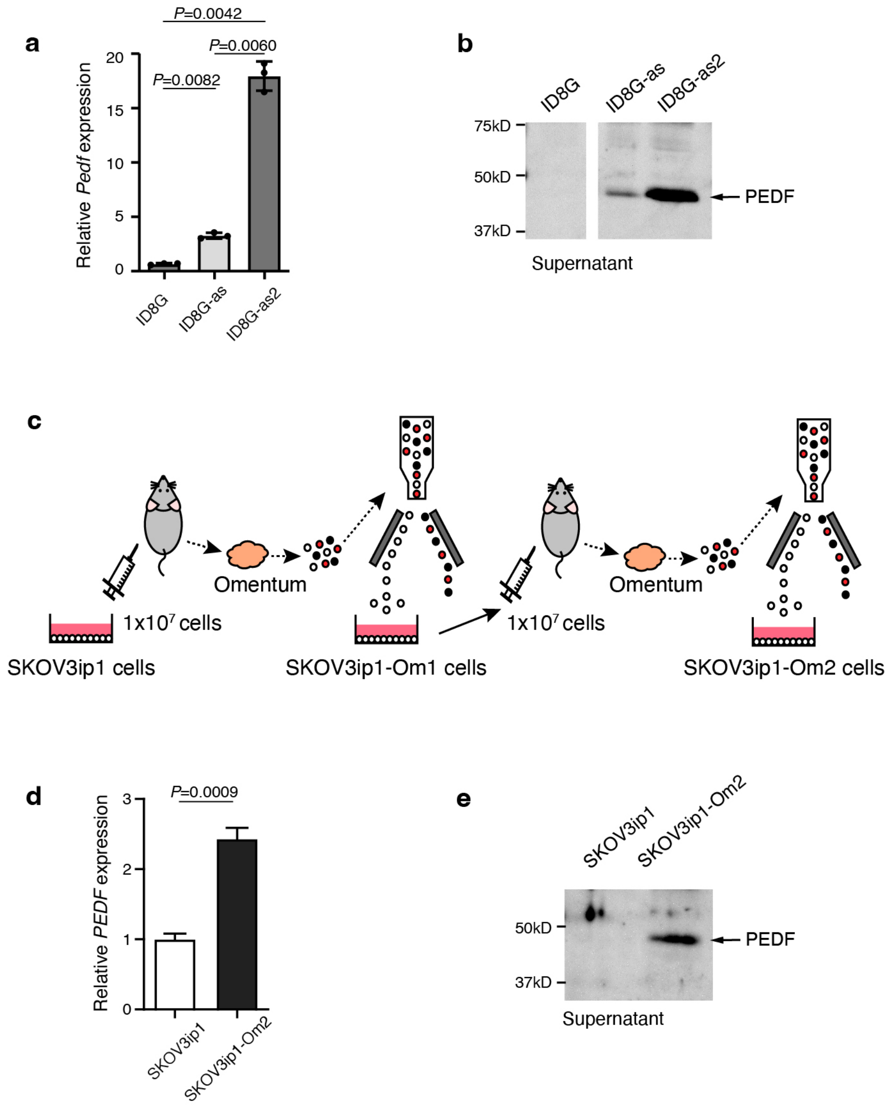

Supplementary Figure 1, related to Fig. 1. a RT-qPCR analysis of PEDF mRNA in ID8G, ID8G-as, and ID8G-as2 cells. **b** Immunoblot analysis of PEDF in culture supernatants of ID8G, ID8G-as, and ID8G-as2 cells. **c** Generation of a human ovarian tumor model based on the injection of EPCAM$^+$ omental tumor cells into the peritoneal cavity. **d** RT-qPCR analysis of PEDF mRNA in SKOV3ip1 and SKOV3ip1-Om2 cells. **e** Immunoblot analysis of PEDF in culture supernatants of SKOV3ip1 and SKOV3ip1-Om2 cells. All quantitative data are means ± SD of three replicates for representative experiments out of a total of three performed. Data for **a** were analyzed by Welch's ANOVA followed by Dunnett's post hoc test, for **d** by unpaired *t* test with Welch's correction.

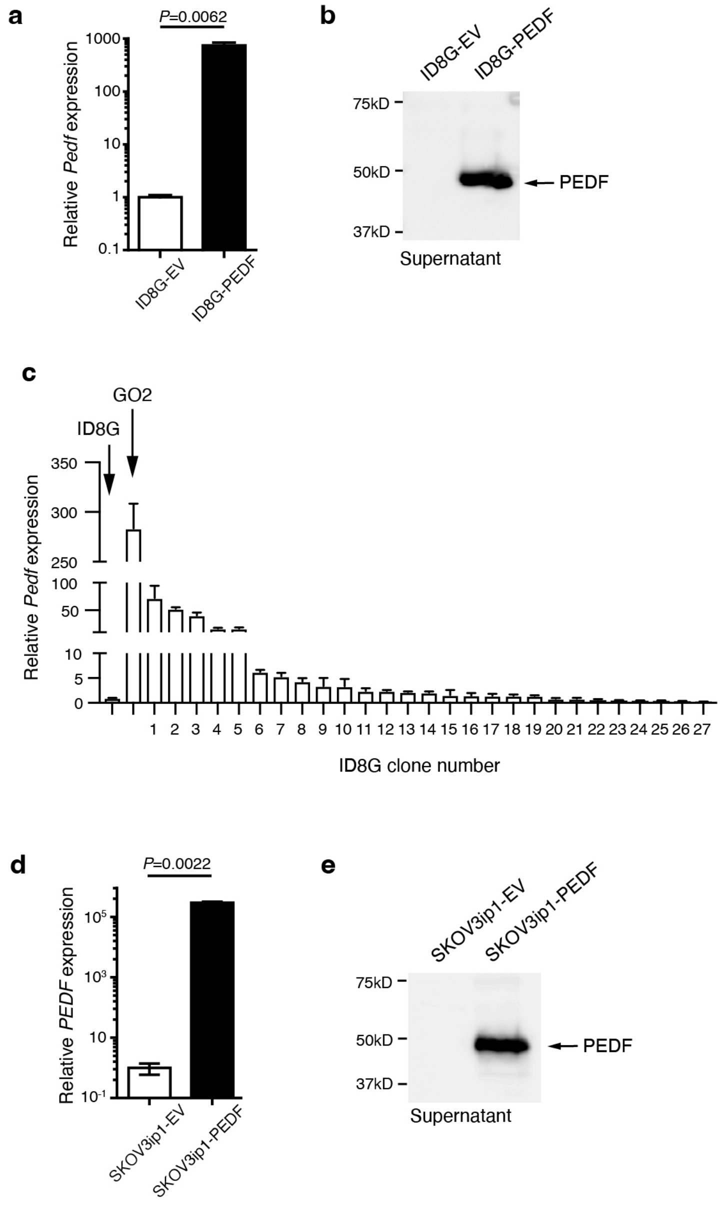

Supplementary Figure 2, related to Fig. 2. a RT-qPCR analysis of PEDF mRNA in ID8G-EV and ID8G-PEDF cells. **b** Immunoblot analysis of PEDF in culture supernatants of ID8G-EV and ID8G-PEDF cells. **c** RT-qPCR analysis of PEDF mRNA in 27 single-cell clones of ID8G cells, relative to parental ID8G cells. **d** RT-qPCR analysis of PEDF mRNA in SKOV3ip1-EV and SKOV3ip1-PEDF cells. **e** Immunoblot analysis of PEDF in culture supernatants of SKOV3ip1-EV and SKOV3ip1-PEDF cells. All quantitative data are means ± SD of three replicates for representative experiments out of a total of three performed and were analyzed by unpaired *t* test with Welch's correction.

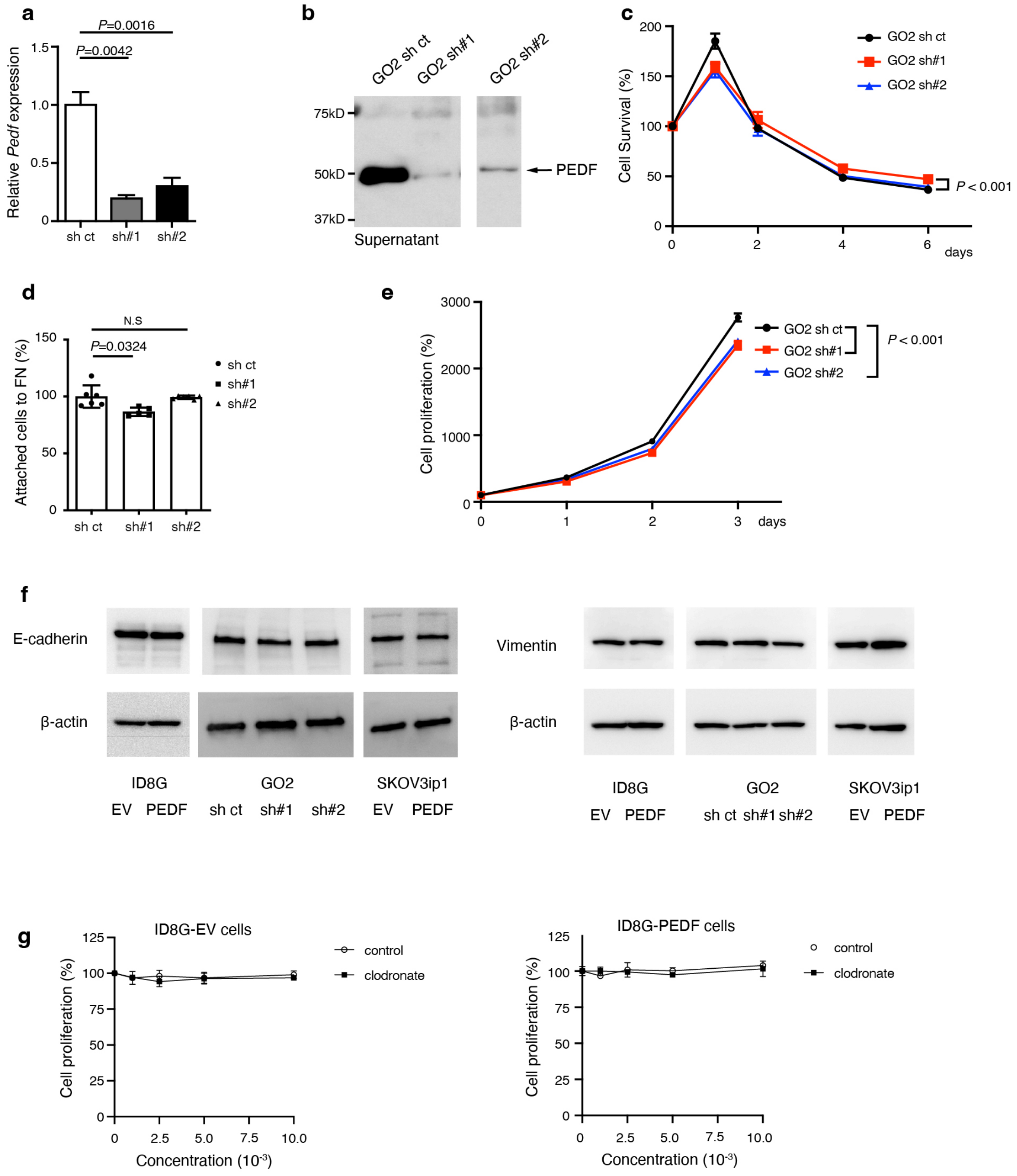

Supplementary Figure 3, related to Fig. 3. a RT-qPCR analysis of PEDF mRNA in GO2 sh#1 or sh#2 or corresponding control (sh ct) cells. **b** Immunoblot analysis of PEDF in culture supernatants of GO2 sh#1 or sh#2 or corresponding control (sh ct) cells. **c** Anoikis resistance assay for cells as in (**a**) (n = 5 each). **d** Attachment assay for GO2 sh#1 or sh#2 or corresponding control (sh ct) cells cultured in wells coated with fibronectin (n = 5 each). **e** Cell proliferation assay for GO2 sh#1 or sh#2 or corresponding control (sh ct) cells (n = 5 each). **f** Immunoblot analysis of E-cadherin

and Vimentin expression. **g** The proliferation of ID8G-EV and ID8G-PEDF cells exposed to clodronate liposomes or control liposomes at a range of concentrations for 48h was assessed (n = 3 each). All data are means ± SD for the indicated number of replicates from representative experiments out of a total of three performed. Data for a were analyzed by unpaired t test with Welch's correction, for **c** and **e** by two-way ANOVA, for **d** by Welch's ANOVA followed by Dunnett's post hoc test.

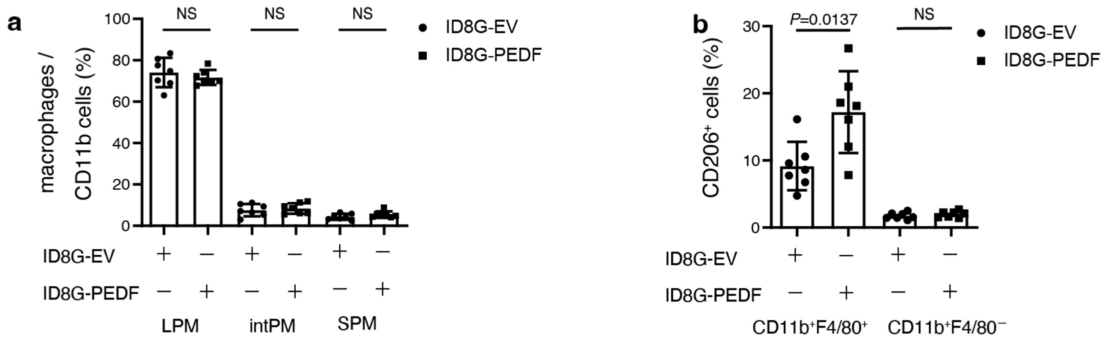

Supplementary Figure 4, related to Fig. 5. (**a**) Percentage of F4/80^{+} MHC II^{-} LPMs, F4/80^{int} MHC II^{int} intPMs, and F4/80^{-} MHC II^{+} SPMs among CD11b^{+} cells in the peritoneal cavity of C57BL/6J mice at 5 days after i.p injection of ID8G-EV or ID8G-PEDF cells (n = 7 mice). (**b**) Percentage of CD206^{+} cells in CD11b^{+}F4/80^{+} cells and CD11b^{+} F4/80^{-} cells in the peritoneal cavity of C57BL/6J mice at 5 days after i.p injection of ID8G-EV or ID8G-PEDF cells (n = 7 mice). Data are means ± SD. *P < 0.05, NS (unpaired t test with Welch's correction).

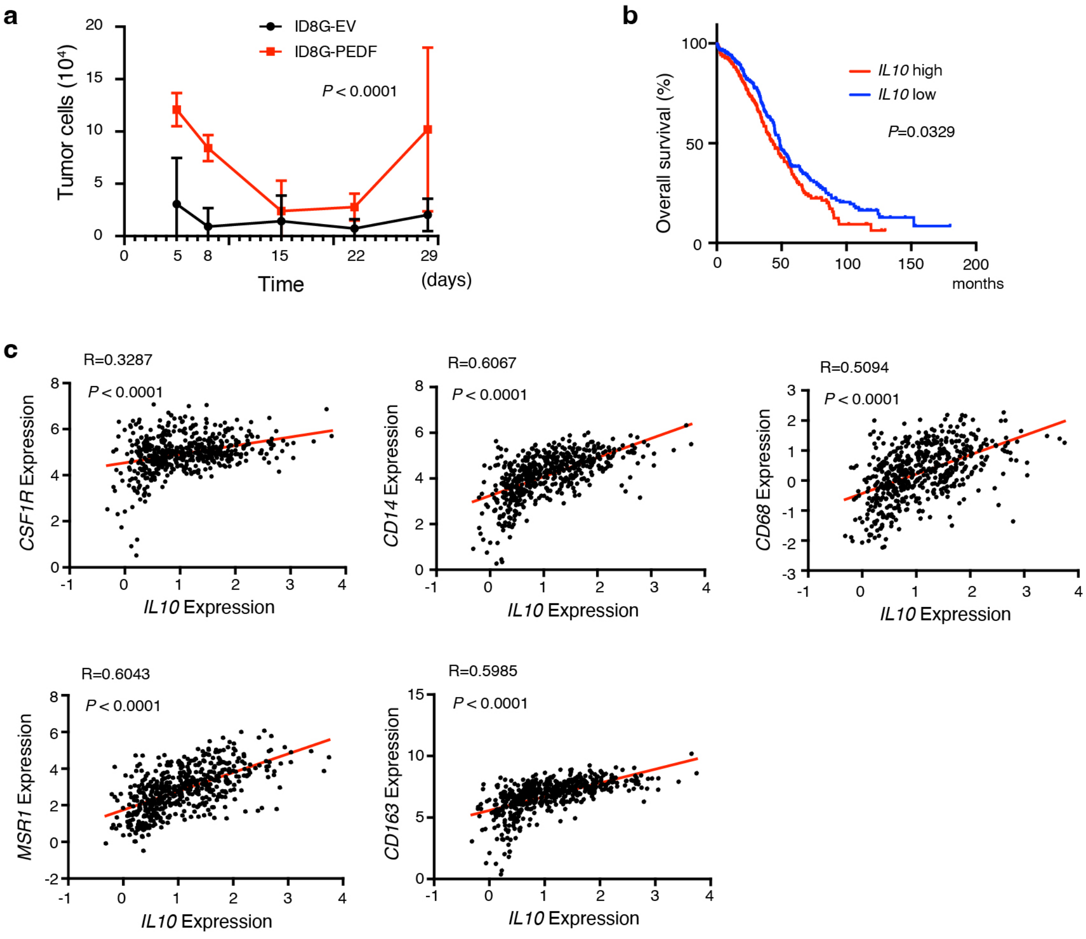

Supplementary Figure 5. (**a**) Number of GFP$^+$ cells in peritoneal washes at the indicated times after i.p. injection of ID8G-EV or ID8G-PEDF cells (5×10^6) into C57BL/6J mice (n = 3 or 4 mice for each group at each time point). At each time point, mice were sacrificed and peritoneal cells were collected for further analysis. Data were analyzed by two-way ANOVA. (**b**) Kaplan-Meier analysis of OS for 529 patients with ovarian serous adenocarcinoma and high or low expression of the *IL-10* in TCGA. The expression cutoff was the median, and the *P* values were determined with the Wilcoxon test. (**c**) Pearson's correlation analysis for expression of *IL10* versus that of *CSF1R, CD14, CD68, MSR1,* or *CD163* in human OC samples of TCGA (n = 531).

Supplementary Figure 6. Uncropped blots for main and supplementary figures.

Full unedited gel for Fig 1g

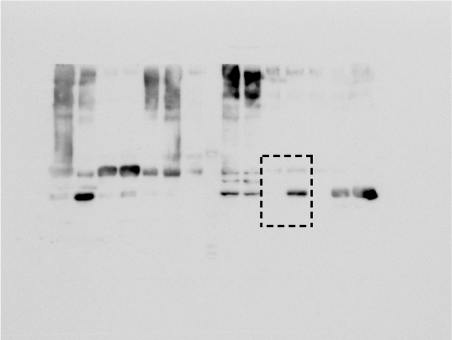

Full unedited gel for Fig 7a

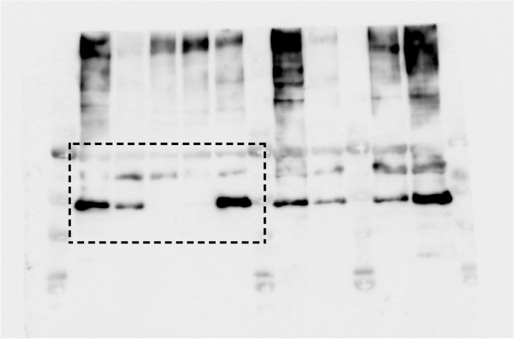

Full unedited gel for Fig 7h
(p53-def-MOSE cells treated with JQ1)

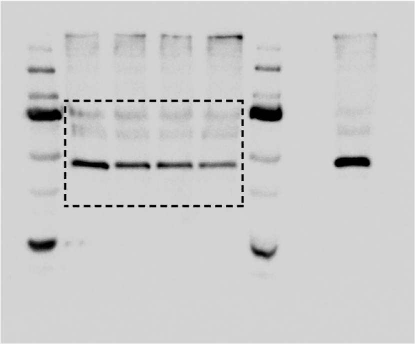

Full unedited gel for Fig 7h
(GO2 cells treated with JQ1)

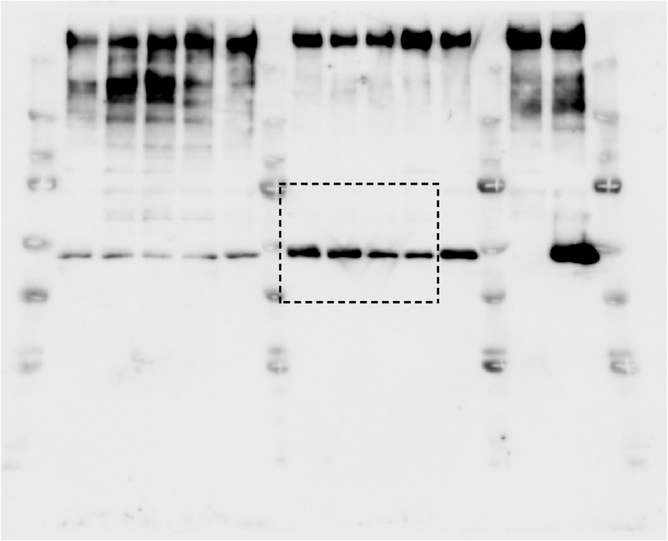

Full unedited gel for Fig 7h
(p53-def-MOSE cells treated with ARV-825)

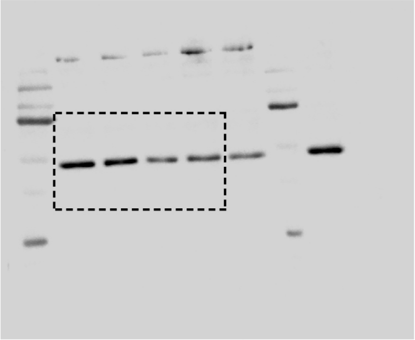

Full unedited gel for Fig 7h
(GO2 cells treated with ARV-825)

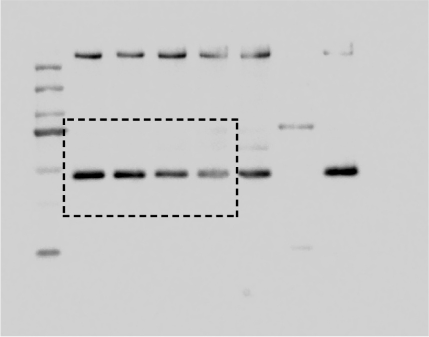

Full unedited gel for Supplementary Fig 1b

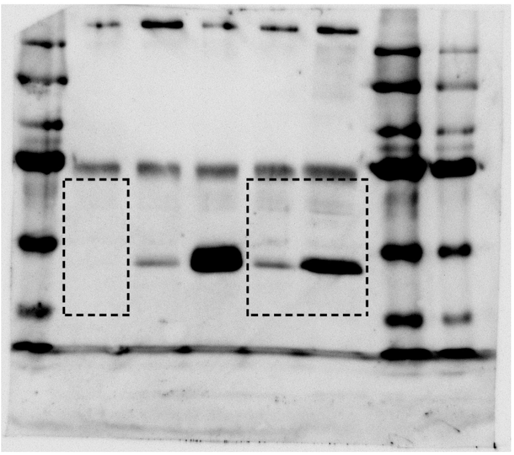

Full unedited gel for Supplementary Fig 1e

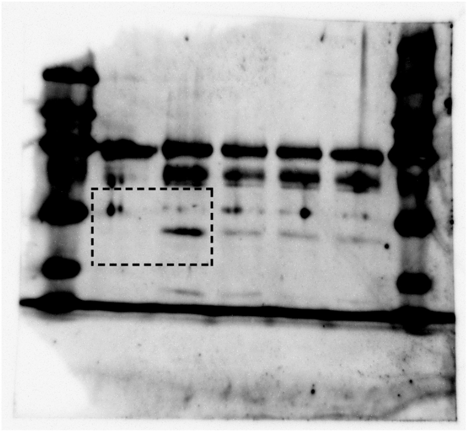

Full unedited gel for Supplementary Fig 2b

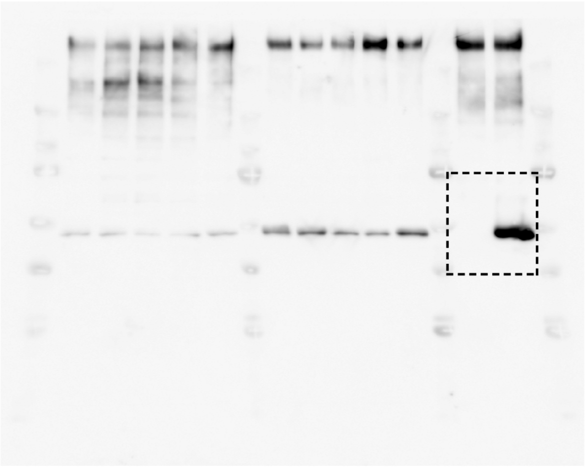

Full unedited gel for Supplementary Fig 2e
(short exposure)

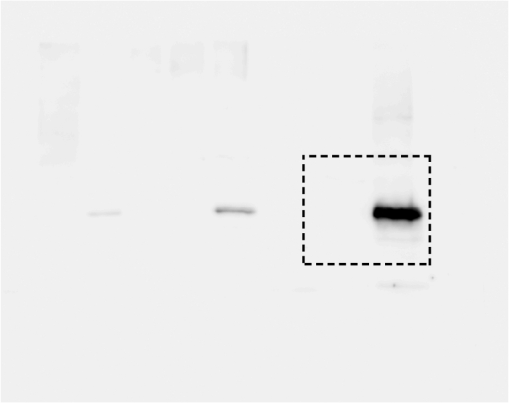

Full unedited gel for Supplementary Fig 2e
(long exposure)

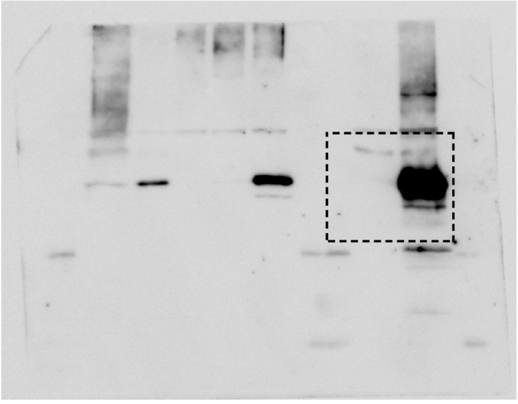

Full unedited gel for Supplementary Fig 3b

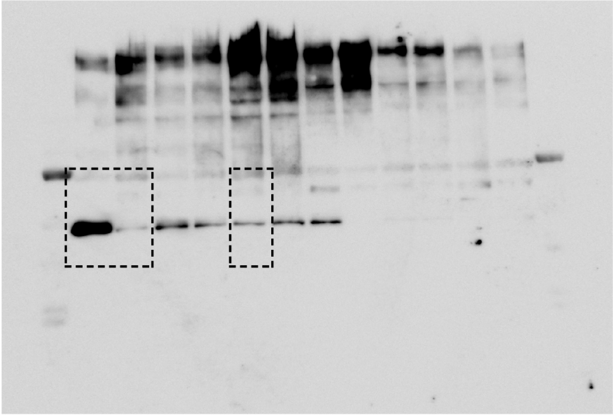

Full unedited gel for Supplementary Fig 3f (E-cadherin)

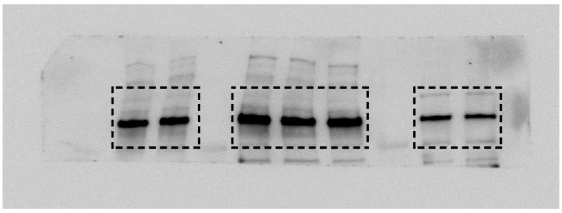

Full unedited gel for Supplementary Fig 3f (β-actin)

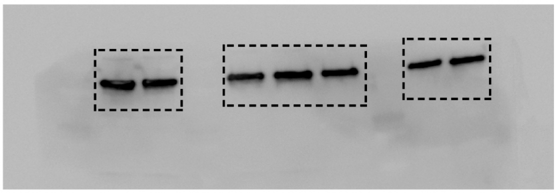

Full unedited gel for Supplementary Fig 3f (Vimentin)

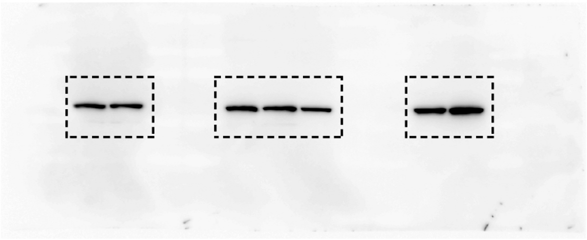

Full unedited gel for Supplementary Fig 3f (β-actin)

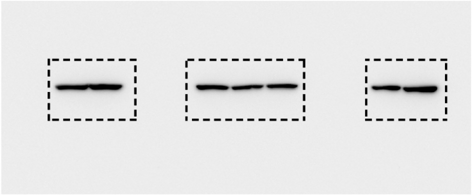

Supplementary Table 1. Kaplan-Meier analysis of overall survival related to expression of genes listed in Fig. 1e.

Gene	Expression	n	OS (months)	*P*
COL8A1	high	264	44.78	0.0656
	low	265	48.75	
CD302	high	262	44.05	0.0126
	low	267	48.75	
DDR2	high	264	44.88	0.0623
	low	265	48.75	
COL1A2	high	265	44.51	0.0851
	low	264	48.72	
FBLN5	high	262	45.34	0.1559
	low	267	47.54	
CASP1	high	264	48.29	0.7270
	low	265	44.78	
APBB1IP	high	263	45.11	0.1042
	low	266	48.72	

Supplementary Table 2. Kaplan-Meier analysis of disease-free survival related to expression of genes listed in Fig. 1e.

Gene	Expression	n	DFS (months)	*P*
COL8A1	high	223	17.87	0.0421
	low	229	18.89	
CD302	high	224	18.04	0.6282
	low	228	17.97	
DDR2	high	223	17.71	0.1798
	low	229	18.96	
COL1A2	high	224	17.58	0.0229
	low	228	19.15	
FBLN5	high	221	18.66	0.3756
	low	231	17.64	
CASP1	high	229	18.20	0.9124
	low	223	17.64	
APBB1IP	high	228	18.00	0.7657
	low	224	17.97	

Supplementary Table 3. Primers used for detection of mRNA expression by RT-qPCR

Genes	Sequences	
	Forward	Reverse
Murine Experiments		
Serpinf1	AAGTTCTGGGTCACGGTCAG	ACGATACGGCTTGGACTCTG
Il10	CGGGAAGACAATAACTGCACCC	CGGTTAGCAGTATGTTGTCCAGC
Mrc1 (CD206)	GTTCACCTGGAGTGATGGTTCTC	AGGACATGCCAGGGTCACCTTT
Actb	CGGTTCCGATGCCCTGAGGCTCTT	CGTCACACTTCATGATGGAATTGA
Human Experiments		
SERPINF1	TGAAGGCGAAGTCACCAAGTCC	CCATCCTCGTTCCACTCAAAGC
ACTB	GGGTCTGGACCTGGCTGGCCGGGACTCG	GGGCCGCCGATCCACACGGAGTACTTGC

Gating information

Fig. 3h, 3i, 4d, 5i, 8b, Sup Fig 5a

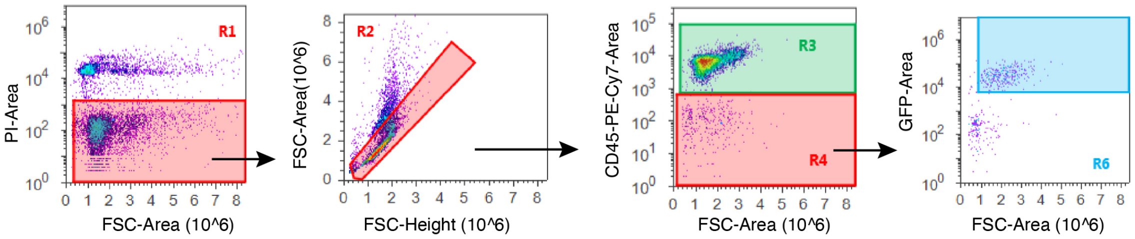

Fig.4e, 8c, 8d

a) CD45 positive cells

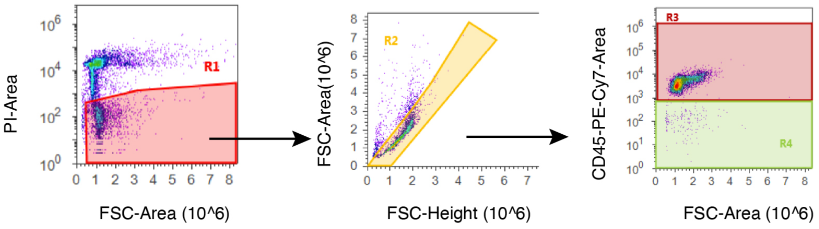

b) CD45 positive CD3 positive cells

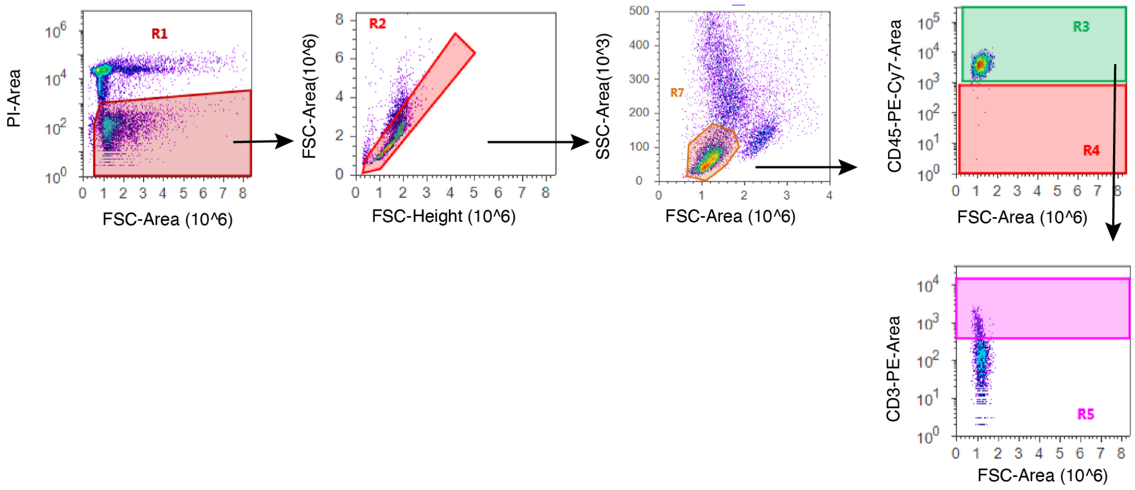

c) CD45 positive CD19 positive cells

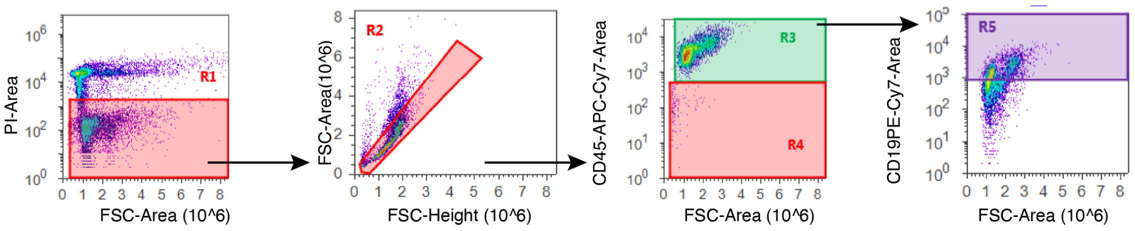

d) CD45 positive CD11b positive cells

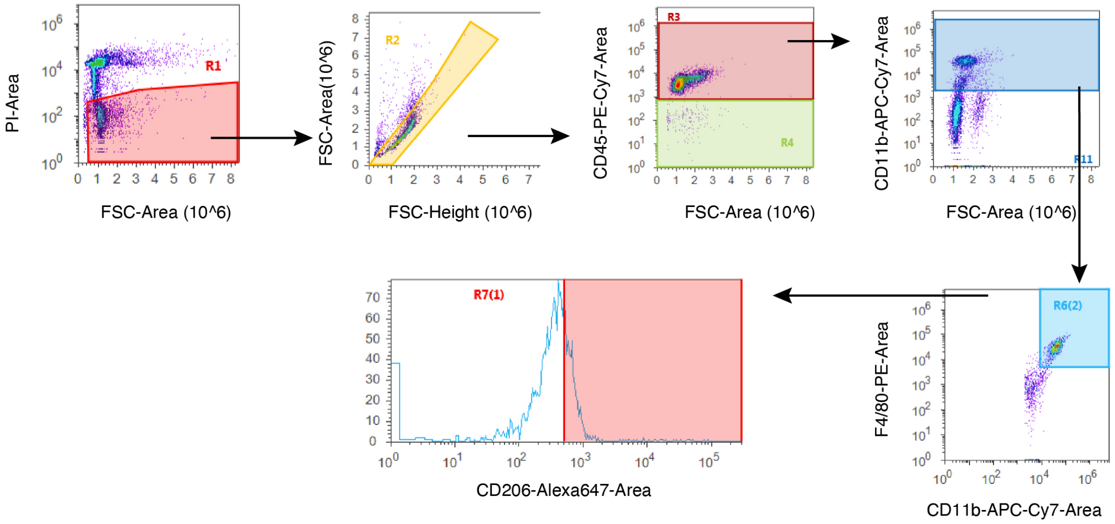

e) CD45 positive CD11c positive cells

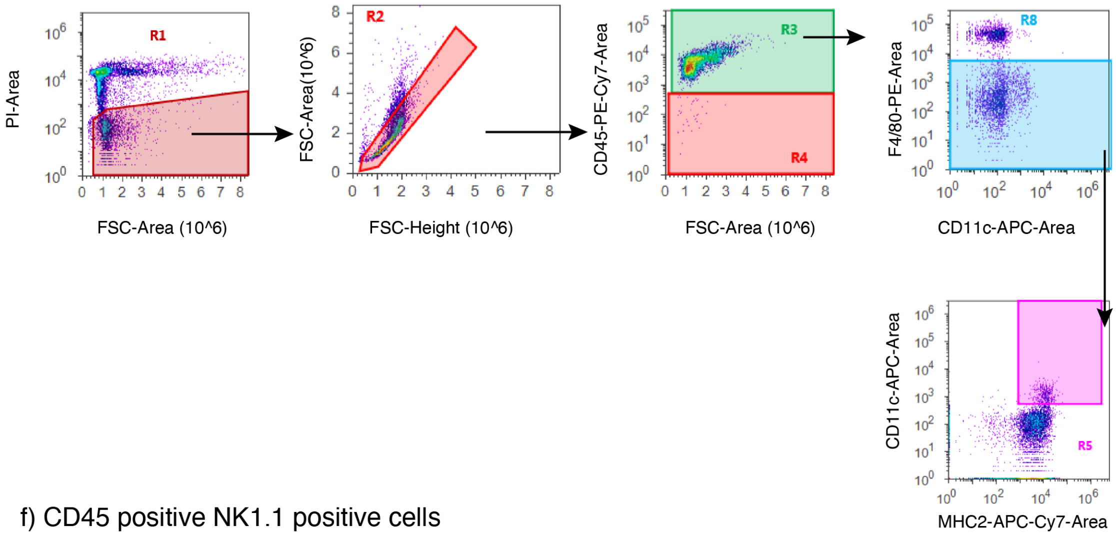

f) CD45 positive NK1.1 positive cells

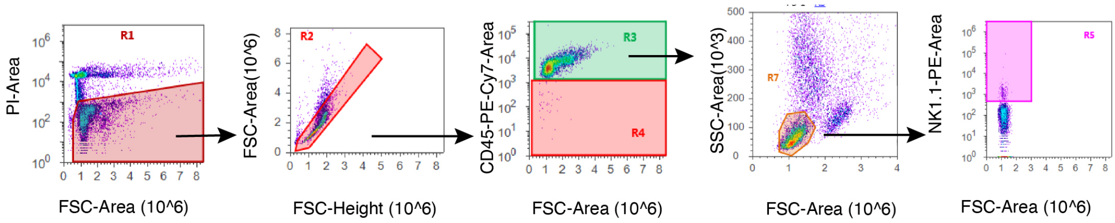

Fig 5b

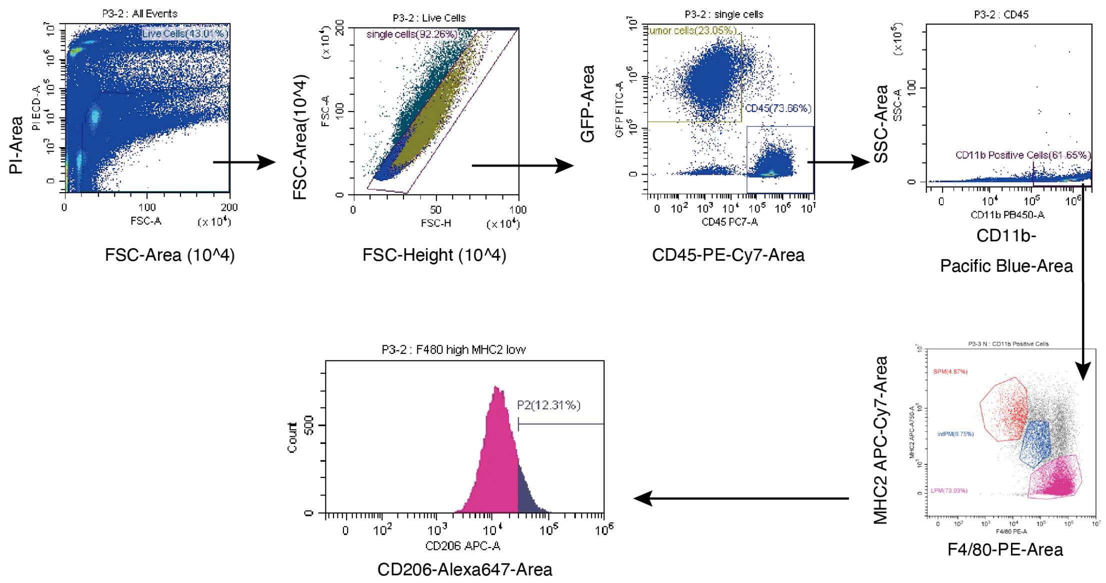

Sup Fig 4a

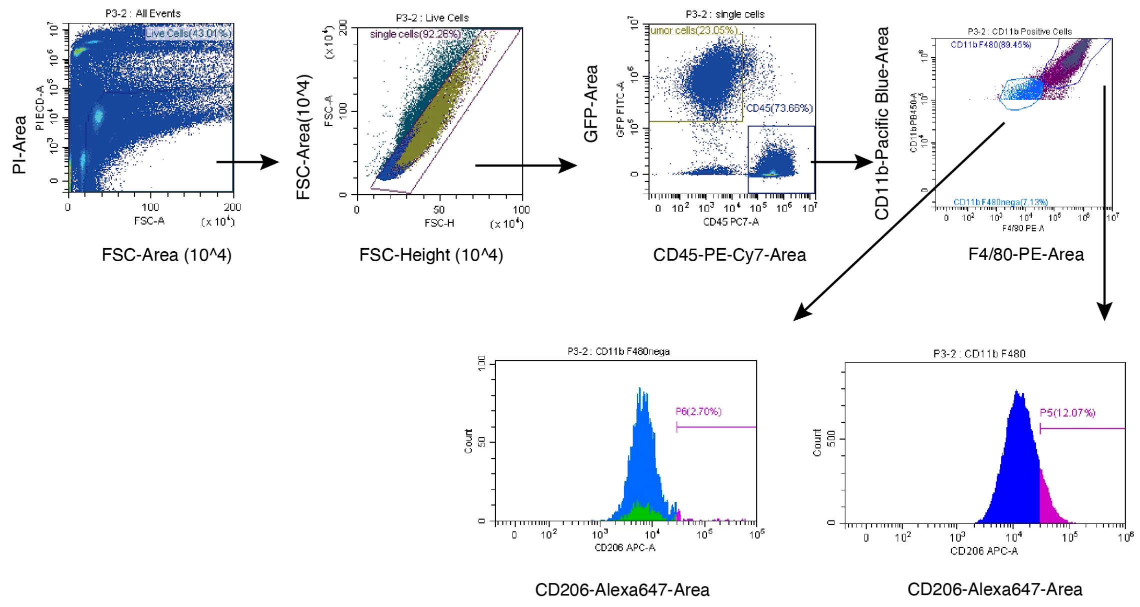

Supplement: Supplementary file 2 — Supplementary Information [file 42003_2022_3837_MOESM2_ESM.pdf]
