# Supplementary material for: Pigment epithelium-derived factor promotes peritoneal dissemination of ovarian cancer through induction of immunosuppressive macrophages
Source: Commun Biol. 2022 Sep 2;5:904. doi: 10.1038/s42003-022-03837-4 (PMC9440245; doi:10.1038/s42003-022-03837-4)
Supplement: Supplementary file 3 — Description of Additional Supplementary Data [file 42003_2022_3837_MOESM3_ESM.pdf]

Description of Additional Supplementary Files

File name: Supplementary Data 1

Description: Microarray Data

File name: Supplementary Data 2

Description: The source data behind the graphs in the paper
