# Supplementary material for: Pigment epithelium-derived factor promotes peritoneal dissemination of ovarian cancer through induction of immunosuppressive macrophages
Source: Commun Biol. 2022 Sep 2;5:904. doi: 10.1038/s42003-022-03837-4 (PMC9440245; doi:10.1038/s42003-022-03837-4)
Supplement: Supplementary file 6 — Reporting Summary [file 42003_2022_3837_MOESM6_ESM.pdf]

nature portfolio

Corresponding author(s): Hideyuki Saya, Eiji Sugihara

Last updated by author(s): Jul 25, 2022

Reporting Summary

Nature Portfolio wishes to improve the reproducibility of the work that we publish. This form provides structure for consistency and transparency in reporting. For further information on Nature Portfolio policies, see our Editorial Policies and the Editorial Policy Checklist.

Statistics

For all statistical analyses, confirm that the following items are present in the figure legend, table legend, main text, or Methods section.

n/a	Confirmed	
☐	☒	The exact sample size (*n*) for each experimental group/condition, given as a discrete number and unit of measurement
☐	☒	A statement on whether measurements were taken from distinct samples or whether the same sample was measured repeatedly
☐	☒	The statistical test(s) used AND whether they are one- or two-sided<br>*Only common tests should be described solely by name; describe more complex techniques in the Methods section.*
☐	☒	A description of all covariates tested
☐	☒	A description of any assumptions or corrections, such as tests of normality and adjustment for multiple comparisons
☐	☒	A full description of the statistical parameters including central tendency (e.g. means) or other basic estimates (e.g. regression coefficient) AND variation (e.g. standard deviation) or associated estimates of uncertainty (e.g. confidence intervals)
☒	☐	For null hypothesis testing, the test statistic (e.g. *F*, *t*, *r*) with confidence intervals, effect sizes, degrees of freedom and *P* value noted<br>*Give P values as exact values whenever suitable.*
☒	☐	For Bayesian analysis, information on the choice of priors and Markov chain Monte Carlo settings
☒	☐	For hierarchical and complex designs, identification of the appropriate level for tests and full reporting of outcomes
☐	☒	Estimates of effect sizes (e.g. Cohen's *d*, Pearson's *r*), indicating how they were calculated

Our web collection on statistics for biologists contains articles on many of the points above.

Software and code

Policy information about availability of computer code

Data collection	-FACS data was collected by either an Attune cytometer, Software version 2.1.0, or CytoFLEX Flow Cytometer, CytExpert Softwear
Data analysis	-FACS data was collected by an Attune cytometer, Software version 2.1.0, or CytoFLEX Flow Cytometer, CytExpert Softwear<br>-Statistical analysis was performed with GraphPad Prism 9.

For manuscripts utilizing custom algorithms or software that are central to the research but not yet described in published literature, software must be made available to editors and reviewers. We strongly encourage code deposition in a community repository (e.g. GitHub). See the Nature Portfolio guidelines for submitting code & software for further information.

Data

Policy information about availability of data

All manuscripts must include a data availability statement. This statement should provide the following information, where applicable:

- Accession codes, unique identifiers, or web links for publicly available datasets
- A description of any restrictions on data availability
- For clinical datasets or third party data, please ensure that the statement adheres to our policy

Human RNA-sequencing data (GSE137237) and microarray data (GSE18521) were obtained from NCBI Gene Expression Omnibus. RNA-sequencing data was analyzed by CLC Gx. CPM (counts per million) was obtained by applying TMM Normalization to the CPM values. TCGA data for patients with ovarian serous cystadenocarcinoma were downloaded from GDC data portal.: https://portal.gdc.cancer.gov/. Data presented in the main figures are available in the Supplementary data. All other data supporting the findings of this study are available either within the body of paper, within the Supplementary information, or are available from the corresponding authors upon reasonable request.

Field-specific reporting

Please select the one below that is the best fit for your research. If you are not sure, read the appropriate sections before making your selection.

☒ Life sciences ☐ Behavioural & social sciences ☐ Ecological, evolutionary & environmental sciences

For a reference copy of the document with all sections, see nature.com/documents/nr-reporting-summary-flat.pdf

Life sciences study design

All studies must disclose on these points even when the disclosure is negative.

Sample size	No statistical method was used to determine sample size. Sample sizes were chosen based on previous experiments.
Data exclusions	No data were excluded from the analysis
Replication	Reproducibility was tested by performing independent replications per experiment as indicated in the manuscript.
Randomization	Animals were randomly allocated to the experimental groups before treatment.
Blinding	No blinding was performed.

Reporting for specific materials, systems and methods

We require information from authors about some types of materials, experimental systems and methods used in many studies. Here, indicate whether each material, system or method listed is relevant to your study. If you are not sure if a list item applies to your research, read the appropriate section before selecting a response.

Materials & experimental systems

n/a	Involved in the study	
☐	☒	Antibodies
☐	☒	Eukaryotic cell lines
☒	☐	Palaeontology and archaeology
☐	☒	Animals and other organisms
☐	☒	Human research participants
☒	☐	Clinical data
☒	☐	Dual use research of concern

Methods

n/a	Involved in the study	
☒	☐	ChIP-seq
☐	☒	Flow cytometry
☒	☐	MRI-based neuroimaging

Antibodies

Antibodies used

anti-mouse CD16/32 (clone 93) BioLegend (San Diego, CA)
anti-mouse CD45 (clone 30-F11) BioLegend (San Diego, CA)
anti-mouse CD19 (clone 6D5) BioLegend (San Diego, CA)
anti-mouse NK1.1 (clone PK136) BioLegend (San Diego, CA)
anti-mouse CD11c (clone N418) BioLegend (San Diego, CA)
anti-mouse CD3ε (clone 145-2C11, Thermo Fisher eBioscience),
anti-mouse CD11b (clone M1/70) BioLegend (San Diego, CA)
anti-mouse F4/80 (clone BM8) BioLegend (San Diego, CA)
anti-I-A/I-E (clone M5/114.15.2) BioLegend (San Diego, CA)
anti-mouse CD206 (ab64693) Abcam
Alexa Fluor 647 anti-rabbit IgG (clone Poly4064) BioLegend (San Diego, CA)

anti-human PEDF (ab14993) for human cell lines
anti-PEDF (ab180711) for mouse cell lines

Validation

All antibodies were validated by the manufacturers.

Eukaryotic cell lines

Policy information about cell lines

Cell line source(s)

ID8 cells were obtained from K. F. Roby (University of Kansas Medical Center).
SKOV3ip1 cells were obtained from D. Yu (University of Texas, MD Anderson Cancer Center).

	p53-def-MOSE and T-Ag-MOSE cells were obtained from JCRB Cell Bank.<br>RAW 264 cells were obtained from RIKEN Cell Bank (Tsukuba, Japan).
Authentication	None of the cell lines used were authenticated.
Mycoplasma contamination	The cell lines were not tested for mycoplasma contamination.
Commonly misidentified lines (See ICLAC register)	No commonly misidentified lines were used in this study.

Animals and other organisms

Policy information about studies involving animals; ARRIVE guidelines recommended for reporting animal research

Laboratory animals	C57BL/6J, female, 6weeks of age were obtained from Oriental Yeast Co., Ltd.<br>BALB/c-nu/nu, female, 5 to 6weeks of age were obtained from Oriental Yeast Co., Ltd.
Wild animals	No wild animals were used in this study.
Field-collected samples	N/A
Ethics oversight	Animal studies were approved by the Animal Care and Use Committee of Keio University School of Medicine (#14031).

Note that full information on the approval of the study protocol must also be provided in the manuscript.

Human research participants

Policy information about studies involving human research participants

Population characteristics	Human serum and ascites samples were provided by the Biobank of Hyogo Cancer Center.
Recruitment	N/A
Ethics oversight	Human part of the study was approved by the Keio University Ethics Committee for Medical Research (#20160388).

Note that full information on the approval of the study protocol must also be provided in the manuscript.

Flow Cytometry

Plots

Confirm that:

☒ The axis labels state the marker and fluorochrome used (e.g. CD4-FITC).

☒ The axis scales are clearly visible. Include numbers along axes only for bottom left plot of group (a 'group' is an analysis of identical markers).

☒ All plots are contour plots with outliers or pseudocolor plots.

☒ A numerical value for number of cells or percentage (with statistics) is provided.

Methodology

Sample preparation	Mouse cells (detailed description available in the methods section)
Instrument	Attune (Thermo Fisher) cytometer or CytoFLEX Flow Cytometer (Beckman Coulter) was used for the flow cytometry analysis
Software	Attune cytometric software version 2.1.0. or CytExpert
Cell population abundance	Cell sorting before flow cytometry analysis was not performed.
Gating strategy	For analysis of alive tumor cell in the peritoneal cavity:<br>Live cell gating: PI negative area from the density plot of FSC-area versus PI<br>Single cell gating: FSC-height versus FSC-area.<br>Analysis: CD45 negative area from the density plot of FSC-area versus PI, followed by GFP positive area from the density plot of FSC-area versus GFP.<br><br>For immune cell analysis using attune cytometric software:<br>Live cell gating: PI negative area from the density plot of FSC-area versus PI<br>Single cell gating: FSC-height versus FSC-area.<br>Analysis:<br>CD45 positive cells; CD45-PE-Cy7 positive area from the density plot of FSC-area versus PE-Cy7.<br>CD45 positive CD11c positive cells; CD45-PE-Cy7 positive, CD11c-APC positive, F4/80-PE negative and MHC2-APC-Cy7 positive cells

CD45 positive CD19 positive cells; CD45-APC-Cy7 and CD19-PE-Cy7 double positive area from the density plot.
CD45 positive CD3 positive cells; CD45-PE-Cy7 and CD3-PE double positive area from the density plot.
CD45 positive NK1.1 positive cells; CD45-PE-Cy7 and NK1.1-PE double positive area from the density plot.
CD45 positive CD11b positive cells; CD45-PE-Cy7 and CD11b-APC-Cy7 double positive area from the density plot.
CD206 positive cells of CD45 positive CD11b positive cells; CD206-Alexa Fluor 647 positive area from histogram.

For immune cell analysis using attune CytExpert:
Live cell gating: PI negative area from the density plot of FSC-area versus PI
Single cell gating: FSC-height versus FSC-area.
Analysis:
LPMs: CD45 positive CD11b positive F4/80 positive MHC2 negative cells; CD45-PE-Cy7 positive, CD11b-Pacific Blue positive, F4/80-PE positive, and MHC2 APC-Cy7 negative area from the density plot.
intPMs: CD45 positive CD11b positive F4/80 positive MHC2 negative cells; CD45-PE-Cy7 positive, CD11b-Pacific Blue positive, F4/80-PE intermediate positive, and MHC2 APC-Cy7 intermediate positive area from the density plot.
SPMs: CD45 positive CD11b positive F4/80 positive MHC2 negative cells; CD45-PE-Cy7 positive, CD11b-Pacific Blue positive, F4/80-PE negative, and MHC2 APC-Cy7 positive area from the density plot.
CD206 positive cells of LPMs, intPMs, SPMs; CD206-Alexa Fluor 647 positive area from histogram.

☒ Tick this box to confirm that a figure exemplifying the gating strategy is provided in the Supplementary Information.
